# Characterization of a Dual CDC7/CDK9 Inhibitor in Multiple Myeloma Cellular Models

**DOI:** 10.3390/cancers5030901

**Published:** 2013-07-24

**Authors:** Alessandro Natoni, Mark R. E. Coyne, Alan Jacobsen, Michael D. Rainey, Gemma O’Brien, Sandra Healy, Alessia Montagnoli, Jürgen Moll, Michael O’Dwyer, Corrado Santocanale

**Affiliations:** 1 Centre for Chromosome Biology, School of Natural Sciences National University of Ireland Galway, Galway, Ireland; E-Mails: alessandro.natoni@nuigalway.ie (A.N.); drmrec@me.com (M.R.E.C.); jafear28@gmail.com (A.J.); michael.rainey@nuigalway.ie (M.D.R.); Gemma.OBrien@nuigalway.ie (G.O.); sandra.healy@nuigalway.ie (S.H.); 2 Department of Medicine, National University of Ireland Galway, Galway, Ireland; 3 Department of Haematology, Galway University Hospital, Galway, Ireland; 4 Nerviano Medical Sciences S.r.l., Via Pasteur 10, Nerviano 20014, Italy; E-Mail: Alessia.Montagnoli@nervianoms.com

**Keywords:** apoptosis, BCL2-family, DNA replication, multiple myeloma, kinase inhibitor, cell cycle

## Abstract

Two key features of myeloma cells are the deregulation of the cell cycle and the dependency on the expression of the BCL2 family of anti-apoptotic proteins. The cell division cycle 7 (CDC7) is an essential S-phase kinase and emerging CDC7 inhibitors are effective in a variety of preclinical cancer models. These compounds also inhibit CDK9 which is relevant for MCL-1 expression. The activity and mechanism of action of the dual CDC7/CDK9 inhibitor PHA-767491 was assessed in a panel of multiple myeloma cell lines, in primary samples from patients, in the presence of stromal cells and in combination with drugs used in current chemotherapeutic regimens. We report that in all conditions myeloma cells undergo cell death upon PHA-767491 treatment and we report an overall additive effect with melphalan, bortezomib and doxorubicin, thus supporting further assessment of targeting CDC7 and CDK9 in multiple myeloma.

## 1. Introduction

Myeloma is a post-germinal B-cell malignancy and the second most common hematological cancer [1]. Despite the use of high dose chemotherapy, autologous stem cell transplantation as well as the recent introduction of immunomodulatory agents and proteasome inhibitors, patients with myeloma have poor outcomes overall, at best only 40% of patients achieve a complete remission and almost all patients eventually relapse and die of their disease [1].

It is generally accepted that the unifying event in myelomagenesis is direct or indirect deregulation of cyclin D, an important mediator of cell cycle control, via non-random primary translocations involving the IgH/L loci [2]. In keeping with the low-proliferation index at diagnosis, deregulated cyclin D alone is insufficient for transformation. Rather, transformation also requires further alteration in known oncogenic drivers (MYC, RAS, P53) or aberrant coactivation and mutually exclusive pairing of G1 cyclin-dependent-kinases (CDKs) -CDK4-cyclin D1 and CDK6-cyclin D2- to occur [2,3]. This leads to a more proliferative and clinically resistant clone where the over-expression of numerous cell cycle and proliferation-related genes can be observed by gene expression profiling (GEP) [4]. Importantly, the patients stratified on the basis of this proliferation signature display the worst overall survival and time to progression [4,5]. These characteristics prompted the investigation of the response of myeloma cells to a range of small molecule CDKs inhibitors. These inhibitors have different specificities, usually targeting various combinations of CDKs [6,7,8,9,10].

The hyper-activation of G1 CDKs can drive cells to transit into S phase and initiate DNA replication from multiple origins that are spread throughout chromosomes [11]. This requires the activity of the cell division cycle 7 (CDC7) serine-threonine kinase. CDC7 is directly involved in the activation of the replicative DNA helicase, the minichromosome maintenance (MCM) complex and in the formation of active replication forks [12,13]. CDC7 phosphorylates several subunits of the MCM complex. Importantly, phosphorylation of MCM2 at serine 40 is completely dependent on CDC7 activity and can be utilized as a specific biomarker of CDC7 activity [14]. CDC7 depletion by small interfering RNA causes numerous tumor cell lines to enter apoptosis in a P53-independent manner while simply arresting cell cycle progression in normal untransformed cells [15,16]. Furthermore, CDC7 depletion, in contrast to replication fork blockade, does not cause a sustained activation of the DNA damage response (DDR) which is a common feature of chemotherapeutics that target DNA replication [16]. These characteristics have led to the development of small molecule inhibitors of the CDC7 kinase with the most advanced currently in phase I clinical studies [17,18].

PHA-767491 is the prototype CDC7 inhibitor and has been shown to have activity in many preclinical cancer models [19]. As well as inhibiting CDC7 kinase this compound, similarly to the more advanced compounds, also potently inhibits the cyclin dependent kinase 9 (CDK9) [19]. This cross-reactivity can be considered beneficial in the treatment of a number of hematological cancers. CDK9 phosphorylates RNA polymerase II (RNA Pol II) and affects the rate of transcription, leading to the depletion in the pool of proteins with short half-lives [20,21]. Among these proteins, the anti-apoptotic protein of the BCL2 family, myeloid cell leukemia-1 (MCL-1), has a critical survival role in most malignant B-cells [10,21,22,23]. Moreover, high expression of MCL-1 and defects in the P53 pathway are now known to be instrumental in inhibiting responsiveness to current and emerging myeloma therapeutics [24,25]. In chronic lymphocytic leukemia (CLL) the cellular responses to the dual CDC7-CDK9 inhibitor PHA-767491 have been studied in some detail and it was observed that these can be greatly influenced by the activation of signaling pathways that confer microenvironmental drug resistance leading to either apoptosis or proliferation arrest [26].

In this study we assessed the activity of PHA-767491 in several cellular models of multiple myeloma including established and primary cell lines and in a co-culture system that partially mimic the bone marrow microenvironment and provides protection against chemotherapeutic drugs currently used in the treatment of this disease [27,28,29]. In these contexts we find that the drug causes cell death and has the potential to overcome the activation of survival signaling pathways.

## 2. Results and Discussion

### 2.1. Cellular Responses of Myeloma Cells to Dual CDC7/CDK9 Inhibitors

In order to understand how myeloma cells respond to CDC7/CDK9 dual inhibition, we initially challenged a panel of human myeloma cell lines with the prototype CDC7/CDK9 inhibitor, PHA-767491. This panel of myeloma cell lines includes the spectrum of primary non-random translocations which are found in myeloma including P53 mutants (Table 1) [30]. Using an ATP-based cell-viability assay, we found that cell viability was lost at low micromolar levels of PHA-767491 in all cell lines with IC_50_ values spanning from 1 to 3.5 μM (Table 1). PHA-767491 is equally active in myeloma cell lines shown to be more resistant to conventional agents including cell lines such as MM1R (dexamethasone), RPMI-8226-Dox40 (doxorubicin) and RPMI-8226-LR5 (melphalan) (Table 1).

**Table 1:** Anti-proliferate activity of PHA-767491 in multiple myeloma cell lines with diverse genetic and molecular features. The indicated established myeloma cell lines were treated with increasing amount of PHA-767491. Cell viability was examined by CellTiter Glo 48 h after drug treatment. The IC_50_ was calculated using GraphPad Prism.

Cell Line	I⁰ Translocation	M-Spike	P53 Status	Copy Number (markers)	PHA-767491 IC50 (μM)
KMS-18	t(4; 14)	IgG lambda	Not Known	1.2 (613)	1
U-266	Insertion IgH switch element on 11q13	IgE lambda	Mutant [31,32]	1.1 (614)	1
OCI-My5	t(14; 16)	Not Known	WT and Mutant [33,34]	2.0 (406)	1.5
RPMI-8226-Dox40	t(14; 16)	IgG lambda	Mutant	Not Known	2
RPMI-8226-LR5	t(14; 16)	IgG lambda	Deficient [32]	Not Known	3.5
MM1S	t(14; 16)	IgG lambda	WT [32]	2.1 (337)	2
MM1R	t(14; 16)	IgG lambda	Not Known	2.2 (336)	1

**Figure 1:**
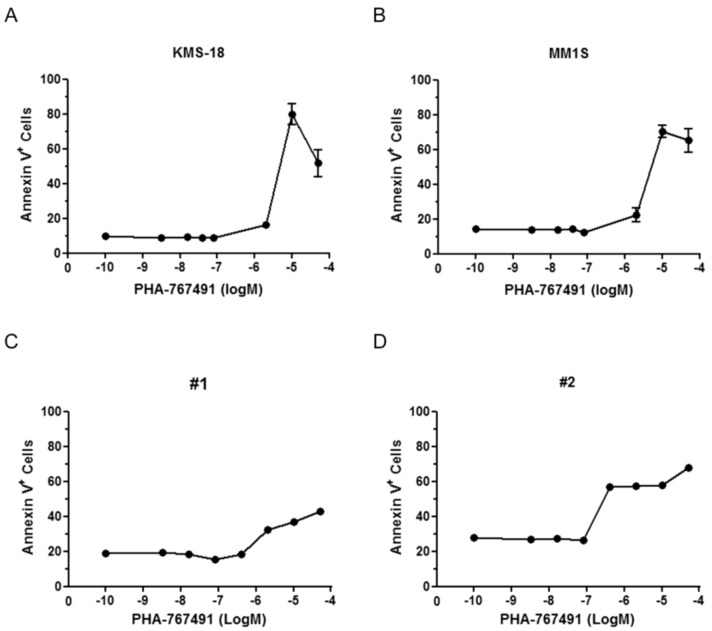
Anti-myeloma activity of PHA-767491. KMS-18 (**A**) and MM1S (**B**) were treated with different doses of PHA-767491 for 24 h and apoptosis was analysed by flow cytometry using AV staining. Data are presented as mean ± standard deviation of two independent repeats; (**C**,**D**) Purified mononuclear cells from two healthy donors were treated with different doses of PHA-767491 for 24 h and apoptosis was analysed by flow cytometry using AV staining. The M in the x axes defines Molarity.

We then tested the activity of PHA-767491 against CD138+ purified primary tumor cells from three myeloma patients who have relapsed after conventional or high-dose cytotoxic chemotherapy as well as novel therapeutics such as immunomodulatory thalidomide derivatives and/or the proteasome inhibitor bortezomib (Table 2). The three patients sampled also represent a cohort with poor prognostic features. PHA-767491 was capable of killing these primary myeloma cells with an average IC_50_ value of 2.3 μM (Table 2). To confirm that loss of ATP measured in the previous experiments was due to cell death and not only to cell cycle arrest, Annexin V staining was performed on KMS-18 and MM1S cells treated with increasing doses of PHA-767491. Twenty-four hours after treatment with active concentrations of the drug most of both KMS-18 and MM1S cells were Annexin V positive indicating that the apoptotic machinery becomes activated in the response of myeloma cells to PHA-767491 (Figure 1A,B). To assess the specificity of PHA-767491 in killing multiple myeloma cells over normal cells, purified mononuclear cells from two healthy donors were treated with increase doses of PHA-767491. The compound was able to kill normal mononuclear cells, however the levels of apoptosis were donor dependent and less than that observed in the multiple myeloma cell lines (Figure 1C,D).

**Table 2:** Anti-proliferate activity of PHA-767491 in multiple myeloma primary samples with diverse genetic and molecular features. Primary myeloma tumor cells isolated from patients were treated with increasing amount of PHA-767491. Cell viability was examined by CellTiter Glo 48 h after drug treatment. The IC_50_ was calculated using GraphPad Prism.

Primary Sample	Cytogenetics	ISS	mSMART	Prior Rx	PHA-767491 IC50 (μM)
GAL-MM-1	Tetraploid, 13q	Stage 3	High Risk	MPT/V/R/D	3
GAL-MM-2	Clonal Evolution. t(11;14)	Stage 3	High Risk	VMPT/ASCT	2
GAL-MM-3	t(4; 14)	Stage 3	High Risk	VRD/ASCT	1.9

### 2.2. PHA-767491 Modulates Biomarkers of CDC7 and CDK9 Activity

**Figure 2:**
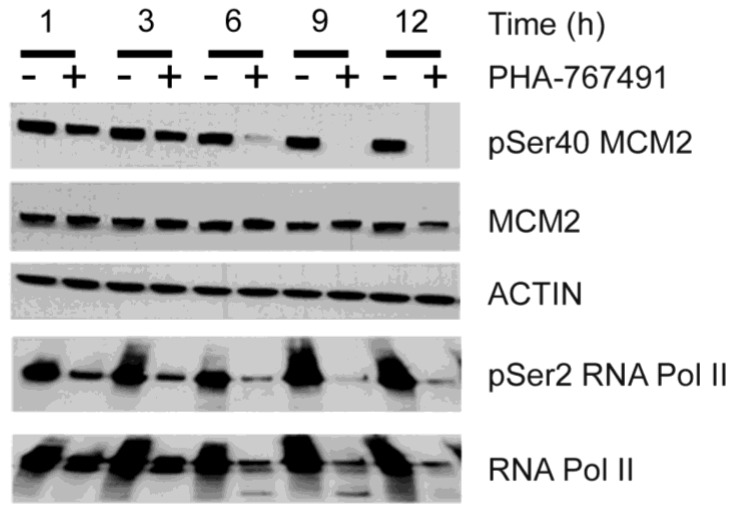
PHA-767491 inhibits both CDC7 and CDK9 in KMS-18 cells. KMS-18 myeloma cells were incubated with 5 μM PHA-767491. Protein extracts were prepared at the indicated time points and analyzed by immunoblotting using the indicated antibodies.

The reduction of Ser40 MCM2 phosphorylation is a sensitive pharmaco-dynamic marker of CDC7 inhibition [19,26,35]. KMS-18 myeloma cells were treated with 5 μM PHA-767491, collected at different times and processed for immunoblotting using specific antibodies recognizing either total MCM2 protein or the CDC7 dependent phosphorylated species of MCM2. Consistent with direct inhibition of CDC7 kinase by PHA-767491, we observed that MCM2 phosphorylation at serine 40 was reduced as early as one hour post-treatment, with a progressive loss at later time points (Figure 2). As a marker of CDK9 inhibition, we measured cellular levels of phosphorylation of the carboxy-terminal repeat domain (CTD) of RNA Pol II at serine 2 [20]. Upon treatment with PHA-767491, levels of pSer2 RNA Pol II decreased together with that of pSer40 MCM2 (Figure 2). These data confirm that PHA-767491 targets both CDC7 and CDK9 in KMS-18 cells.

### 2.3. MCL-1 Is Potently Downregulated Following PHA-767491 Treatment

**Figure 3:**
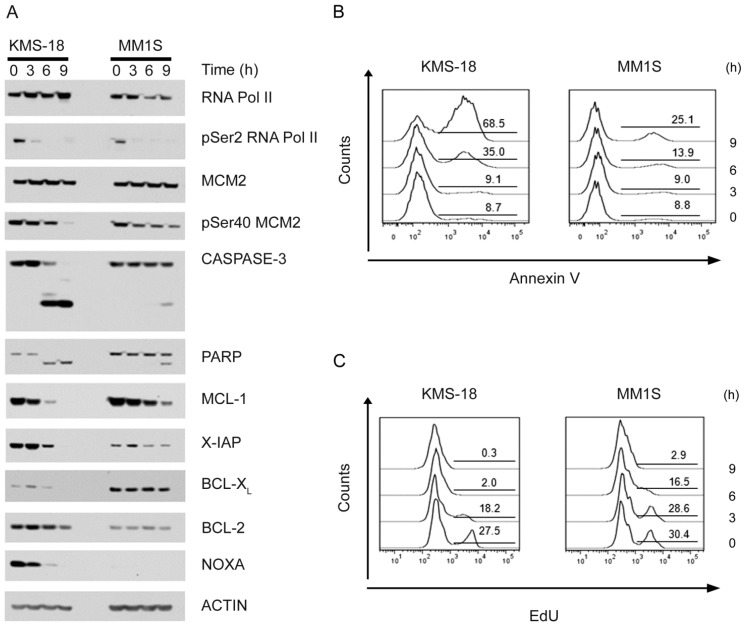
Analysis of pro- and anti-apoptotic proteins in response to PHA-767491. KMS-18 and MM1S myeloma cells were incubated with 5 μM PHA-767491 for the indicated time. Protein extracts were prepared and analyzed by immunoblotting using the indicated antibodies (**A**). In parallel samples, apoptosis (**B**) and DNA synthesis (**C**) were analysed by flow cytometry using AV staining and EdU incorporation assay respectively. Numbers in the gated regions represent the percentage of cells positive for either AV (**B**) or EdU (**C**) staining.

**Figure 4:**
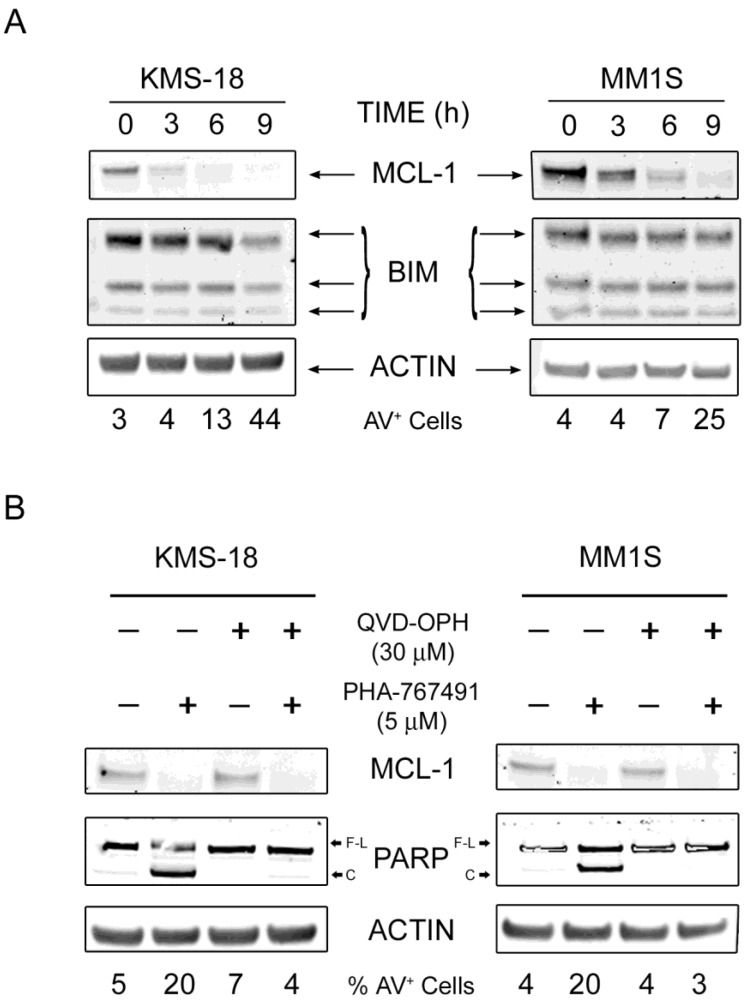
PHA-767491 does not affect BIM levels and causes caspase-independent MCL-1 downregulation. (**A**) KMS-18 and MM1S myeloma cells were incubated with 5 μM PHA-767491 for the indicated time. Protein extracts were prepared and analyzed by immunoblotting using the indicated antibodies; (**B**) KMS-18 and MM1S cells were incubated with 30 μM QVD-OPH for 30 min and then treated with 5 μM PHA767491for 6 h (KMS-18) or 9 h (MM1S). After incubation, protein extracts were prepared and analyzed by immunoblotting using the indicated antibodies. F-L and C in the PARP lane indicate full length and cleaved respectively. Percentage of AV^+^ cells is indicated at the bottom.

We have previously shown that in CLL primary cells, PHA-767491 induces apoptosis through the intrinsic pathway most likely by downregulating MCL-1 protein levels and that upregulation of BCL-X_L_ upon CD40 stimulation can limit apoptotic cell death [26]. In order to gain a better understanding of the molecular determinants of PHA-767491-induced apoptosis in myeloma cells, we analysed the levels and dynamics of several pro- and anti-apoptotic proteins in both KMS-18 and MM1S cells in a time course experiment after exposure to 5 µM PHA-767491. Samples were also taken to assess DNA synthesis and levels of apoptosis by measuring the percentage of cells incorporating the thymidine analogue EdU in their DNA and exposing phosphatidylserine on the outer membrane respectively. The levels of pSer2 RNA Pol II decreased very rapidly following PHA-767491 treatment in both cell lines (Figure 3A). PHA-767491 treatment also diminished the levels of pSer40 albeit with slower kinetics compared to pSer2 RNA Pol II (Figure 3A). PHA-767491 induced a marked and rapid downregulation of MCL-1 proteins levels in KMS-18 which was rather slow and less pronounced in MM1S at these early time points (Figure 3A). Downregulation of MCL-1 levels in KMS-18 correlated with a robust caspase-3 activation, PARP cleavage and loss of X-IAP. Again, these events were less evident in MM1S, supporting a direct correlation between MCL-1 downregulation and caspase activation. Moreover, the appearance of AV^+^ cells in both cell lines and a complete chase of DNA synthesis were observed and these effects occurred faster and were more pronounced in KMS-18 than in MMS1 cells (Figure 3B,C), although at later time points the number of apoptotic cells was equivalent for both cell lines. The percentage of AV^+^ cells directly correlated with the relative amount of cleaved PARP and cleaved caspase 3 detected in the extracts and inversely correlated with the levels of anti-apoptotic protein MCL-1. The relative levels of the anti-apoptotic protein BCL2 were higher in KMS-18 than MM1S and did not change significantly during the time of treatment (Figure 3A). Intriguingly the pro-apoptotic protein NOXA, that normally binds and counteracts MCL-1 [36], was undetectable in MM1S cells and was downregulated in KMS-18 following PHA-767491 treatment arguing against a role of NOXA in PHA-767491-induced apoptosis in myeloma cells. Instead, the levels of BCL-X_L_ were higher in MM1S than KMS-18 and were not affected by the PHA-767491 treatment (Figure 3A). To better understand the mechanism(s) of action of PHA-767491, the protein levels of BIM which together with NOXA represents the major binding partner of MCL-1 [36], were analysed in both KMS-18 and MM1S cells in a time course experiment after exposure to 5 µM PHA-767491. Again, PHA-767491 induced a marked downregulation of MCL-1 which was accompanied by an increase in AV^+^ cells, however the protein levels of BIM were stable with a slight decrease in KMS-18 at 9 h post-treatment probably due to the high levels of apoptosis (Figure 4A). Importantly, the decrease in the MCL-1 protein levels were not a consequence of caspase activation as inhibition of caspases by the broad spectrum caspase inhibitor QVD-OPH did not prevent downregulation of MCL-1 although completely prevented phosphatidylserine exposure and PARP cleavage (Figure 4B). Altogether these results suggest that the downregulation of MCL-1 contributes to PHA-767491-induced apoptosis.

### 2.4. Effect of Stroma Cells on the Response of Myeloma Cells to PHA-767491

**Figure 5:**
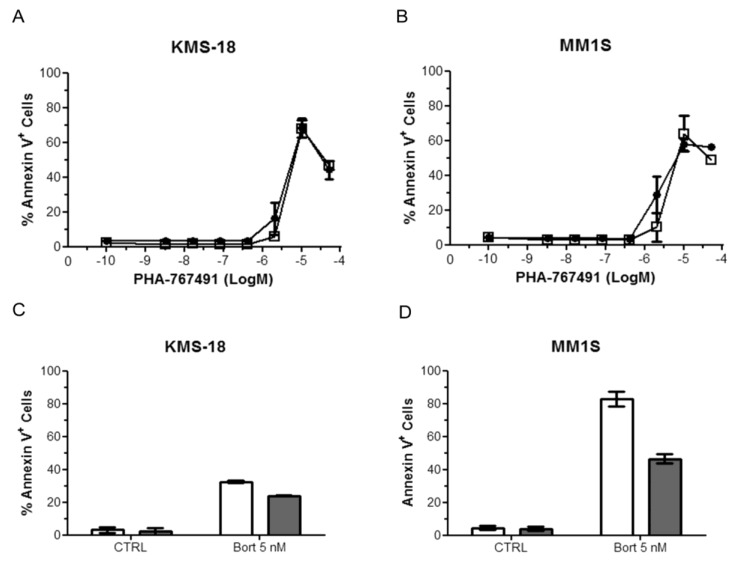
PHA-767491 induces apoptosis in KMS-18 and MM1S in co-culture with HS5 cells. KMS-18 (**A**) and MM1S (**B**) cells were cultured with (open square) or without (black circle) HS5-H2B-GFP cells for 2 h and then treated with different doses of PHA-767491 for a further 24 h. Apoptosis was assessed by AV staining. In parallel samples, KMS-18 (**C**) and MM1S (**D**) cultured with (gray bars) or without (white bars) HS5-H2B-GFP cells for 2 h, were treated with 5 nM bortezomib and apoptosis was analysed 24 h after treatment using AV staining. Data are presented as mean ± standard deviation of two independent repeats. The M in the x axes defines Molarity.

Given the role that microenvironmental signals play in drug resistance [37], we tested the apoptotic inducing activity of PHA-767491 in KMS-18 and MM1S cells in a co-culture system with HS5 cells, a model that partially mimics the bone marrow microenvironment [38]. To be able to discriminate HS5 from multiple myeloma cells, HS5 cells were firstly engineered to express the green fluorescent protein (GFP)-tagged Histone H2B (HS5-H2B-GFP). Multiple myeloma cells were then plated on HS5-H2B-GFP cells for 2 h and then treated with different doses of PHA-767491 for a further 24 h; apoptosis was assessed by AV/PI staining as described in Material and Methods. Bortezomib at 5 nM was used to assess the HS5-H2B-GFP-mediated drug resistance. HS5-H2B-GFP cells did not protect KMS-18 and MM1S from PHA-767491-induced apoptosis (Figure 5A,B) although they reduced bortezomib-induced apoptosis, in particular in MM1S cells (Figure 5C,D).

### 2.5. PHA-767491 Shows an Additive Effect with Melphalan, Bortezomib and Doxorubicin

**Figure 6:**
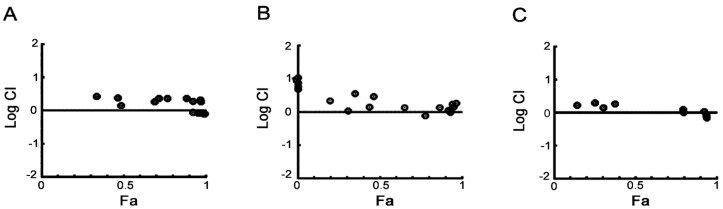
Combination Analysis of PHA-767491 using Chou-Talalay Median-Effect Equation. MM1S cells were treated with PHA-767491 alone and in combination with Melphalan (Range 2 to 32 µM, **A**), Doxorubicin (Range 31 to 500 nM, **B**) or Bortezomib (Range 1.3 to 20 nM, **C**) in a non-constant ratio in a simultaneous fashion. Cell viability was examined by CellTiter Glo 48 h after drug treatment. Combination indices (CI) were calculated and the Log CI (circles) plotted in relation to the fraction of cells affected (Fa) at any given experimental point.

**Figure S1:**
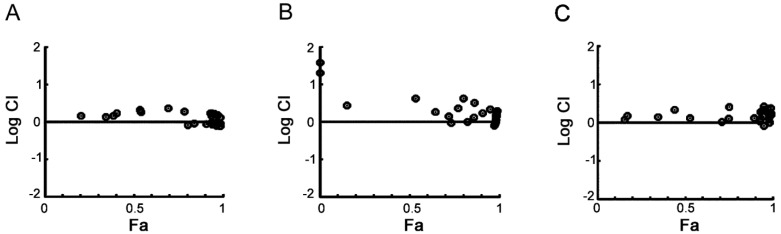
Sequential combination analysis of PHA-767491 with drugs constituting standard of care. MM1S cells were treated with PHA-767491 for 3 h followed by treatment with Melphalan (Range 2 to 32 µM, **A**) Doxorubicin (Range 31 to 500 nM, **B**) Bortezomib (Range 1.3 to 20 nM, **C**) in a non-constant ratio. Cell viability was examined by CellTiter Glo 48 h after drug treatment. Combination indices (CI) were calculated and the Log CI (circles) plotted in relation to the fraction of cells affected (Fa) at any given experimental point.

**Figure S2:**
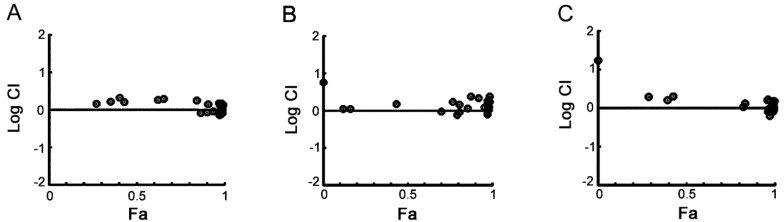
Sequential combination analysis of PHA-767491 with drugs constituting standard of care. MM1S cells were treated with Melphalan (Range 2 to 32 µM, **A**) Doxorubicin (Range 31 to 500 nM, **B**) Bortezomib (Range 1.3 to 20 nM, **C**) for 3 h followed by treatment with PHA-767491 in a non-constant ratio. Cell viability was examined by CellTiter Glo 48 h after drug treatment. Combination indices (CI) were calculated and the Log CI (circles) plotted in relation to the fraction of cells affected (Fa) at any given experimental point.

In order to test the potential of a CDC7/CDK9 dual inhibitor in combination with standards of care, MM1S cells were treated with PHA-767491 together with melphalan, bortezomib, or doxorubicin. Combination experiments were performed by treating cells with each drug in constant and non-constant combination ratios. After incubation, cell viability was assessed and data was then analyzed with the Chou-Talalay median-dose-effect formula. The combination index (CI) for each experimental combination point was calculated [39]. In the first set of experiments, drugs were administered simultaneously. We observed that almost all CI calculated were either equal or very similar to 1 (Figure 6). Similarly, when the drugs were combined in a sequential manner with either melphalan, bortezomib or doxorubicin administered 3 h before or after PHA-767491, the calculated CI was still very close to 1 (Supplementary Figure S1 and Figure S2). Overall, these results indicate a general additive cytotoxic effect of PHA-767491 with each standard of care in the myeloma cell line used for these experiments.

### 2.6. Discussion

PHA-767491 is the first nanomolar, ATP-competitive, CDC7 small-molecule inhibitor that has been broadly characterized as an anticancer agent in preclinical models [19]. A growing number of compounds that inhibit CDC7 are either in preclinical development or in the initial stages of clinical development [17,18,19,40,41,42]. An exciting feature of CDC7 inhibition is the fact that it induces a reversible cell cycle arrest in normal primary cells while inducing apoptosis in a P53-independent manner in many different cancer cell lines [16,19]. The underlying reason for this differential effect is yet to be fully understood, but is likely to be due to defects in the checkpoint pathways that control transition through the different phases of the cell cycle including P53 and other tumor suppressor genes such as FOXO3a, P15/INK4 and DKK3 [15] as well as loss of the equilibrium between pro-apoptotic and anti-apoptotic proteins [26].

Together with CDC7 kinase, PHA-767491 also targets CDK9. Cross reactivity with CDK9 is also maintained in the more advanced CDC7 inhibitors [41,42], suggesting a structural similarity in the active sites of these two kinases, thus this new class of drugs can be defined as dual CDC7/CDK9 inhibitors.

Key issues for investigators working with dual CDC7/CDK9 inhibitors are to discriminate the cellular effects caused by these two concomitant inhibitory activities in cellular assays and the identification of potential target diseases that are most likely to respond to this treatment strategy.

Multiple lines of evidence suggest that hematologic malignancies are highly dependent on survival signaling proteins and disruption of the relative equilibrium of anti-apoptotic and pro-apoptotic proteins is sufficient to drive these cells into apoptosis [7,22,25,43,44]. In a recent work we have shown that in chronic lymphocytic leukemia (CLL), the cellular responses to PHA-767491 are highly context dependent. The compound causes apoptosis in quiescent cells isolated form peripheral blood independent of prognostic markers, and while able to block DNA synthesis it is less efficient in promoting apoptosis of the same CLL cells when these are stimulated into proliferation in a system that mimics the lymph node microenvironment [26].

In this work, we show that the dual CDC7/CDK9 inhibitor, PHA-767491, kills myeloma cell lines including P53 mutant cells and cell lines that have developed resistance to dexamethasone, melphalan and doxorubicin. Furthermore, PHA-767491 has the same effect on primary myeloma cells harvested from patients who have relapsed with progressive refractory disease. This suggests that the mechanisms leading to chemoresistance to conventional chemotherapy in myeloma may not affect the activity of a dual CDC7/CDK9 inhibitor. Unlike in our previous study in CLL, mechanistically we were unable to separate the two inhibitory activities either in time course or dose response experiments and therefore the relative contribution of CDC7 and CDK9 inhibition in driving cell death in this specific setting still needs to be determined. However we provide some circumstantial evidence that, as in CLL, the expression of MCL-1 is likely to be important in the survival of myeloma cells to PHA-767491. Moreover, the slower kinetics of PHA-767491-induced apoptosis observed in MM1S might be related to high levels of BCL-X_L_ in this cell line compared to KMS-18. This is consistent with a recent work showing that BCL-X_L_ is a key determinant of resistance to apoptosis for those compounds that, like PHA-767491, induce apoptosis by repressing MCL-1 expression levels [45]. Further experiments are required to sustain this hypothesis. The downregulation of NOXA observed in KMS-18 cells may be related to transcriptional inhibition directly caused by PHA-767491 or it may reflect its accelerated degradation through the recently described ubiquitin-independent pathway that is blocked by MCL-1 [46,47]. We also found mild cytotoxic effects of PHA-767491 in primary mononuclear cells purified from two healthy donors. This result was not surprising as transient lymphopenia is a common side effect of CDK9 inhibitors in the clinic and may be a consequence of a reduction in cellular levels of MCL-1 due to its transcriptional inhibition [48].

The importance of the microenvironment in supporting myeloma growth and survival has been demonstrated previously [49,50]. This has led to the establishment of technical platforms that permit assessment of the contribution of multiple factors, including direct interaction with stromal cells, to multiple myeloma cell response to chemotherapeutic agents in a high throughput manner [37]. In this study, we observed that co-culturing the multiple myeloma cells with HS5-H2B-GFP stromal cells does not confer resistance to PHA-767491-mediated apoptosis although the co-culture partially protects from bortezomib-induced apoptosis, suggesting that the apoptotic inducing activity of PHA-767491 might not be affected by the stromal microenvironment.

Finally, as CDC7 inhibitors progress in the clinic, the ability to devise rational combinations will become of importance. Our *in vitro* experiments indicate that PHA-767491 has an overall additive effect when combined with melphalan, bortezomib, and doxorubicin in an *in vitro* setting and therefore, there is the potential for this new drug class to be included in combination regimens.

## 3. Experimental Section

### 3.1. Chemicals

PHA-767491 was from Nerviano Medical Sciences (Nerviano, Italy), bortezomib was from OrthoBiotech (Horsham, PA, USA); doxorubicin, melphalan, dexamethasone and QVD-OPH were from Sigma-Aldrich (St. Luis, MO, USA). Recombinant human insulin-like growth factor-1 and interleukin-6 were from R&D (Minneapolis, MN, USA). 5-Ethylnyl-2'-deoxyuridine (EdU) and 6-carboxy-fluorescein-TEG azide were from Berry & Associates (Dexter, MI, USA). Fluorescein isothiocyanate (FITC, Molecular Probes, Eugene, OR, USA)-conjugated Annexin V was prepared in house as previously described [51]. Allophycocyanin (APC)-conjugated Annexin V was from Immunotool (Friesoythe, Germany).

### 3.2. Cell Culture

The multiple myeloma cell lines KMS-18, OCI-My5, and U266, were kindly provided by Dr Marta Chesi, Dr Leif Bergsagel and Dr Keith Stewart (Mayo Clinic, Scottsdale, AZ, USA). RPMI-8226-LR5 and RPMI-8226-Dox 40 were kindly provided by Dr William Dalton (Moffitt Cancer Center, Tampa, FL, USA). MM1S and MM1R were kindly provided by Dr Steven Rosen (Northwestern University, Chicago, IL, USA). All multiple myeloma lines were cultured in RPMI 1640 media (Sigma-Aldrich) supplemented with 10% fetal bovine serum (Sigma-Aldrich), 2 mM L-glutamine (Sigma-Aldrich), 50 U/mL penicillin (Sigma-Aldrich), and 50 µg/mL streptomycin (Sigma-Aldrich). The HS5 cells were from ATCC. A variant of HS5 cells expressing the Histone H2B tagged with the Green Fluorescent Protein (HS5-H2B-GFP) was generated by stably transfecting the HS5 cells with pBOS-H2B-GFP construct and sorting the GFP positive cells. The HS5-H2B-GFP cells were grown in DMEM (Sigma-Aldrich) supplemented with 10% fetal bovine serum, 2 mM L-glutamine (Sigma-Aldrich), 50 U/mL penicillin (Sigma-Aldrich), and 50 µg/mL streptomycin (Sigma-Aldrich). All cell lines were maintained in a state of logarithmic growth at 37 °C in humidified air with 5% CO_2_.

Myeloma patient and healthy donor samples were obtained with informed consent. This was carried out with approval of the local governing Ethics Committee in accordance with the Declaration of Helsinki. Bone marrow mononuclear cells were separated using Ficoll-Hypaque density sedimentation, and plasma cells were purified (>95%, CD138+) by positive selection with anti-CD138 MACS Microbeads (Miltenyl, Bisley, UK). Peripheral blood mononuclear cells from healthy donors were purified by Ficoll-Hypaque density sedimentation.

For co-culture experiments, HS5-H2B-GFP cells were plated at 5 × 10^4^ cells/well in 48 well/plate and incubated for 48 h. After incubation, multiple myeloma cells were plated at 5 × 10^5^ cell/mL (0.5 mL/well) in presence/absence of HS5-H2B-GFP. The cells were then incubated for 2 h and then treated with relevant drugs for a further 24 h.

### 3.3. Cell Viability Assay

Cells were seeded in triplicate at a density of 5,000 and 10,000 cells in 100 μL in 96 well plates, treated with drug(s) and analyzed 48–72 h post treatment with a cell viability assay—Cell TitreGlo (Promega, Madison, WI, USA). IC_50_ (Median effect [D_m_]) was calculated with both the Chou-Talalay based median-effect equation and a non-linear regression four parametric logistic graph-fitting approach (slope, IC_50_, upper and lower value normalization) using Compusyn (Compusyn Inc., Paramus, NJ, USA) [39] and GraphPad Prism (GraphPad Software Inc., La Jolla, CA, USA) respectively. The combination between PHA-767491 and other anti-myeloma agents (doxorubicin, melphalan, and bortezomib) was analyzed with Compusyn software [39].

### 3.4. Immunoblotting

Cells were lysed in lysis buffer [1% sodium dodecyl sulfate (SDS)] or TGN buffer [Tris (pH 8.0), 150 mmol/L NaCl, 1.0% Tween 20, 10% glycerol, 1 mmol/L phenylmethylsulfonyl fluoride (PMSF), 5 µg/mL aprotinin, 5 µg/mL leupeptin, 75 mmol/L NaF, 20 mmol/L ß-glycerolphosphate, 0.4 mmol/L sodium vanadate, and 1 mmol/L DTT]. Proteins were separated by SDS-polyacrylamide gel electrophoresis (SDS-PAGE), transferred to nitrocellulose membranes and analyzed by western blotting. Antibodies against MCL-1, total CASPASE-3, PARP were purchased from Cell Signaling (Danvers, MA, USA). Antibodies against BCL2 (clone 100), BCL-X_L_ (clone H-5) were from Santa Cruz Biotechnology (Dallas, TX, USA). The antibody against BIM was from Enzo Life Sciences (Farmingdale, NY, USA). Antibodies against MCM2 were raised against the N-terminus of human MCM2 protein in collaboration with Pocono Rabbit Farm and Laboratory Inc. (Canadensis, PA, USA). Anti-pSer40/41 MCM2 antibody was as previously described [14]. Antibodies against RNA Pol II and pSer2 RNA Pol II were purchased from Covance Research Products (Princeton, NJ, USA).

### 3.5. DNA Replication and Apoptosis Assays by Flow Cytometry

For DNA replication assay, cells (1 × 10^6^) were incubated with 10 μmol/L EdU for 1 h, collected, washed with PBS, fixed in 2% paraformaldehyde for 5 minutes, washed with PBS and resuspended in 1 mL permeabilization buffer [0.5% w/v saponin in 1% (w/v) BSA/PBS]. For click reaction, 10 mmol/L Na-L-ascorbate, 100 μmol/L 6-carboxyfluorescein-TEG azide, and 2 mmol/L CuSO_4_ were added sequentially. Samples were incubated for 30 minutes at room temperature in the dark, followed by addition of 10 volumes of 1% (w/v) BSA in 0.5% (v/v) Tween 20/PBS and incubated for a further 10 minutes. After three washes, samples were resuspended in PBS and analyzed with BD FACSCanto I (BD Biosciences, Franklin Lakes, NJ, USA). Annexin V-FITC assay was performed as previously described [26]. Apoptosis in the context of the HS5-H2B-GFP co-culture was assessed by staining the cells with Annexin V APC-conjugated and gating on the GFP negative cells.

## 4. Conclusions

In the present study we have shown that the prototype of an emerging class of kinase inhibitors targeting both CDC7 and CDK9 kinases has cell death inducing activity in myeloma cellular models. This together with the additive effects observed when PHA-767491 is used in combination with approved drugs currently used in chemotherapeutic regimens for MM treatment, suggests that further investigation into CDC7/CDK9 inhibition in this disease is warranted.
